# Evaluation of Immunohistochemical Marker Bcl-2 in Epithelial Dysplasia and Squamous Cell Carcinoma of the Oral Cavity

**DOI:** 10.7759/cureus.66576

**Published:** 2024-08-10

**Authors:** Sonali Choudhury, Surekha U Arakeri, Lathadevi HT

**Affiliations:** 1 Department of Pathology, Shri BM Patil Medical College Hospital and Research Center, Vijayapura, IND; 2 Department of Otorhinolaryngology, Shri BM Patil Medical College Hospital and Research Center, Vijayapura, IND

**Keywords:** squamous cell carcinoma, oral cavity, dysplastic lesions, b-cell lymphoma gene 2, anti-apoptotic gene

## Abstract

Introduction: Squamous cell carcinoma of the oral cavity is the most common malignancy noted globally.Pathogenesis of premalignant and malignant oral lesions is mainly attributed to the alteration in the molecular mechanisms that regulate apoptosis and cell proliferation. B-cell lymphoma gene 2 (Bcl-2) is the anti-apoptotic gene that prolongs cell survival by inhibiting apoptosis and is associated with the aggressive behaviour of malignant tumours. The aim of the study was to evaluate Bcl-2 expression in oral squamous cell carcinoma and dysplastic lesions of the oral cavity and to compare its expression with various grades of dysplasia and carcinoma.

Methodology: A hospital-based cross-sectional study was done on 80 clinically suspected cases of dysplastic and malignant oral cavity lesions received in the histopathology section of the Department of Pathology of Shri BM Patil Medical College Hospital and Research Center, Vijaypura, Karnataka. Out of 80 cases, 40 were squamous cell carcinoma, and 40 were dysplastic lesions of the oral cavity. For each case, two sections measuring 4 μm thickness were prepared. Hematoxylin and eosin staining was performed on one section, and Bcl-2 IHC staining was performed on another. Bcl-2 expression evaluation was done for each case of oral epithelial dysplasia and squamous cell carcinoma.

Results: Out of 40 cases of squamous cell carcinoma, 15 were well-differentiated, 22 were moderately differentiated, and three were poorly differentiated. In well-differentiated oral squamous cell carcinoma, Bcl-2 positivity was grade 0 in 66.7% of cases and grade 1 in 33.3% of cases. In moderately differentiated oral squamous cell carcinoma, Bcl-2 positivity was grade 1 in 63.6% of cases and grade 2 in 36.4% of cases. In poorly differentiated oral squamous cell carcinoma, Bcl-2 positivity was grade 1 in 33.3% and grade 2 in 66.7% of cases. Out of 40 cases of dysplastic lesions, 11 cases showed severe dysplasia, 11 cases showed moderate dysplasia and 18 cases showed mild dysplasia. grade 2 positivity was seen in 72.7% of cases of severe dysplasia and 63.6 % of cases of moderate dysplasia. In mild dysplasia, all of the cases showed grade 0 Bcl-2 expression.

Conclusion: In poorly differentiated oral squamous cell carcinoma Bcl-2 positivity was high and low in well-differentiated oral squamous cell carcinoma. Bcl-2 expression was higher in severe dysplasia compared to moderate dysplasia, which may indicate aggressive behaviour of tumour in poorly differentiated oral squamous cell carcinoma and severe dysplasia.

## Introduction

Squamous cell carcinoma of the oral cavity is one of the most frequent cancers reported in India, which makes up 95% of all malignancies seen in the oral cavity. Oral premalignant lesions like leucoplakia, erythroplakia, lichen planus, mixed white and red lesions, and verrucous lesions have the potential to transform into malignancy, and their potential for transformation is associated with the degree of dysplasia they exhibit [1,2]. Treatment for oral malignancy is decided based on features such as the size of the tumour, distant metastases, and lymph node involvement. However, these criteria, in some cases, could not justify the poor prognosis in patients of carcinoma who were diagnosed at an early stage [2].

Death of normal and malignant cells occurs by a process of apoptosis. The Bcl-2 family of proteins controls this phenomenon. Bcl-2 is an anti-apoptotic prosurvival protein that is overexpressed in many cancer cells. This may cause dysregulation of the apoptotic mechanism, lead to increased survival of the cells, and cause resistance to therapeutic intervention [3].

A high level of Bcl-2 expression was observed in various haematological malignancies, such as chronic lymphocytic leukaemia, diffuse large B cell lymphoma and mantle cell lymphoma. It is also overexpressed in solid tumours such as carcinoma of the lung, breast and gliomas. Some studies observed aberrantly decreased levels of pro-apoptotic proteins such as BH3, which initiate apoptosis. Based on this observation, novel anticancer drugs called BH3 mimetics are developed, which activate the apoptosis mechanism by inhibiting the prosurvival Bcl-2 protein. BH3 mimetics, such as venetoclax, is used in clinical trials of chronic lymphocytic leukaemia and acute myeloid leukaemia. Thus, Bcl-2 expression studies in various malignancies may help develop Bcl-2 inhibitor drugs as a novel therapy for cancer [4].

In some of the recent studies, it was mentioned that Bcl-2 family proteins have become major therapeutic targets in various malignancies. Many treatment options based on the regulation of Bcl-2 family proteins are expected [5]. In breast carcinoma, Bcl-2 expression has inconsistent results. Some studies mentioned that Bcl-2 overexpression has adverse effects, and some studies have mentioned that Bcl-2 overexpression has better disease-free survival in some subtypes of carcinoma breast [6].

Positive Bcl-2 expression and distribution have been linked in several studies to the more aggressive behaviour of oral malignant tumours, which suggests a poor clinical outcome. This information could help us understand the biological behaviour of premalignant and dysplastic oral lesions, particularly cancerous lesions, which could have significant therapeutic and prognostic implications [1,2]. Hence, the present study evaluated the expression of the Bcl-2 marker in the oral cavity's dysplastic lesions and squamous cell carcinoma.

## Materials and methods

Source of data: This study was done in the Department of Pathology, Bijapur Lingayat District Education (Deemed to be University) Shri B M Patil Medical College, Hospital and Research Centre, Vijayapura, Karnataka, India. It is a hospital-based cross-sectional study. Over 21 months, from September 2022 to May 2024, 80 cases were selected. In this study, 40 cases were oral squamous cell carcinoma (OSCC), and 40 were oral epithelial dysplasia (OED). Ethical clearance was obtained from the Institutional Ethical Committee for this study (IEC/672/2022-23 dated 30.08.2022).

Inclusion and exclusion criteria: Biopsy and resection specimens of clinically suspected premalignant and malignant oral cavity lesions diagnosed with dysplasia and malignancy on histopathology were included in the study. Tissues that were inadequate for further processing for immunohistochemistry were excluded. Biopsy specimens of patients on radiotherapy and chemotherapy were excluded.

Data collection methods: All specimens underwent tissue processing per standard protocol, and paraffin blocks were prepared. Two 4 μm sections were prepared for every case. Hematoxylin and eosin (H&E) staining was performed on one section, while Bcl-2 immunohistochemical staining was performed on another. Cases that underwent histopathological diagnosis of OSCC and OED were included in the study. Tissue sections were placed on pre-coated slides and incubated in an incubator for one hour at 60°C. The slides were deparaffinized by keeping 30 minutes in an incubator set at 60°C. For antigen retrieval, sections were stored in citrate buffer and microwaved. After adding 3% hydrogen peroxide, the slide was left for ten minutes. Then, the sections were washed using 0.05 mM Tris-buffered saline solution with pH 7.4. Diluted monoclonal antibodies targeting Bcl-2 were utilized as primary antibodies. Primary antibodies were applied to these sections and incubated for one hour at 37°C. Tris-buffered saline solution (0.05 mM) was used for washing. Dextran polymers labelled with peroxidase were used to conjugate the secondary antibody. After that, sections were treated with a 0.5 mg/ml solution of 3,3'-diaminobenzidine that contained 0.001% hydrogen peroxide. Mayer's hematoxylin was used for counterstaining. Slide mounting was completed after sections were cleaned in xylene and dried in ethanol. Tonsillar tissue was used as a positive control.

For OSCC, Bcl-2 expression was considered positive when both membrane and cytoplasmic staining by Bcl-2 was noted. Sections with invasive tumour areas were selected by screening at 100x magnification. Then, under 400X magnification, Bcl-2 expression scoring was done. It was ensured that the selected area had at least 100 tumour cells. For OED, Bcl-2 positivity was studied in various layers of the lining epithelium, including the basal, parabasal, and superficial layers. Sections with dysplastic areas were selected by screening at 100X magnification and under 400X magnification, and Bcl-2 expression scoring was done. After counting the Bcl-2 positive tumour cells, the grading was done as Grade 0 (negative expression) - 0-10% of tumour cells showing Bcl-2 positivity, Grade 1 (low expression) - 10% to 30% of tumour cells showing Bcl-2 positivity, Grade 2 (moderate expression) - 30% to 60% of tumour cells showing Bcl-2 positivity and Grade 3 (high expression) - more than 60% of tumour cells showing Bcl-2 positivity [2]. Bcl-2 expression scoring was done similarly for dysplastic lesions, as mentioned above.

Sample size: The formula used to calculate sample size was as per the study by Arya et al. [2]. The required minimum sample size was 80 to achieve a power of 80% for detecting differences in proportions between two groups at a two-sided p-value of 0.05.

Statistical analysis of the data was done using JMP-SAS software (https://www.jmp.com/). The results were presented in a Microsoft Excel sheet. Percentages and mean ± SD were calculated. The association between Bcl-2 expression in OED and OSCC was analyzed using the Chi-square test for statistical significance. All statistical tests performed were two-tailed. A p-value of less than 0.05 was considered statistically significant.

## Results

Eighty cases were included in the current study, out of which 40 were OSCC while the remaining 40 were OED. Resection specimens received were three, and biopsies were 77. The maximum number of cases of OSCC was found in the age group of 51-60 years, amounting to 12 cases(30%), with the majority of the cases being males, amounting to 27 cases (68%), and the male-female ratio was 2.08:1 (Table 1).

**Table 1:** Distribution of age and sex in OSCC (n=40) OSCC- Oral squamous cell carcinoma

Age (in years)	Males		Females		Total	
	N	%	N	%	N	%
21-30	3	11.1%	1	7.6%	4	10%
31-40	3	11.1%	0	00%	3	7.5%
41-50	6	22.3%	2	15.3%	8	20%
51-60	7	25.9%	5	38.4%	12	30%
61-70	4	14.8%	2	15.3%	6	15%
71-80	4	14.8%	3	23.4%	7	17.5%
Total	27	100%	13	100%	40	100%

**Figure 1:**
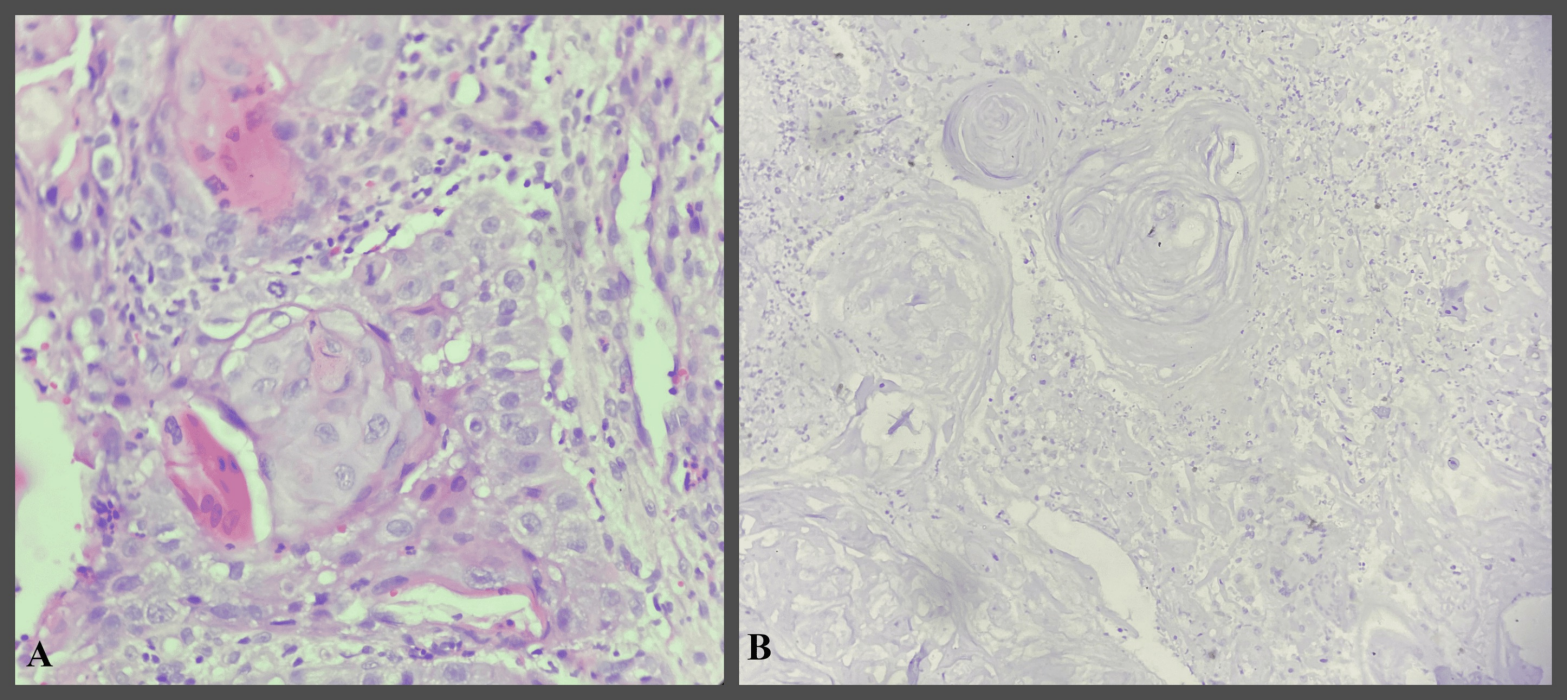
IHC study images showing well-differentiated OSCC OSCC- Oral squamous cell carcinoma H&E- Hematoxylin and Eosin IHC- Immunohistochemical A- H&E stain 400x  B- Bcl-2 IHC stain 400x showing Grade 0 positivity

**Figure 2:**
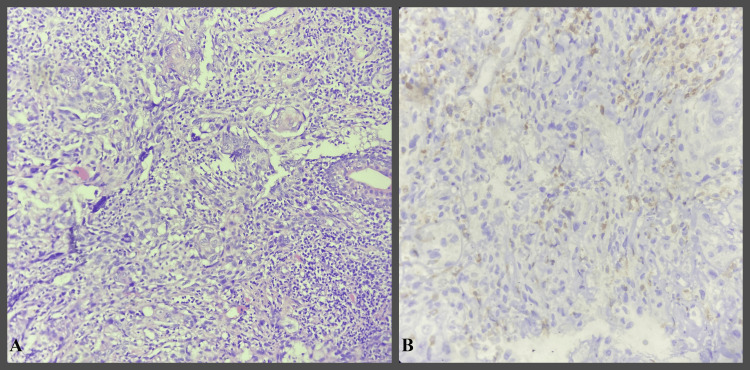
IHC study images showing moderately differentiated OSCC OSCC - Oral squamous cell carcinoma H&E- Hematoxylin and Eosin IHC- Immunohistochemical A- H&E stain 400x  B- Bcl-2 IHC stain 400x - showing Grade 2 positivity

**Figure 3:**
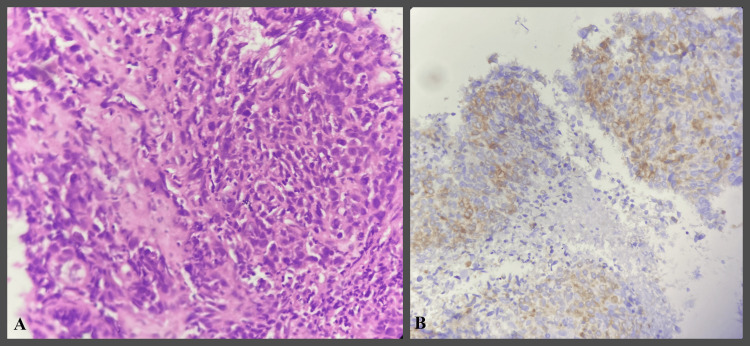
IHC study images showing poorly differentiated OSCC OSCC- Oral squamous cell carcinoma H&E- Hematoxylin and Eosin IHC- Immunohistochemical A- H&E stain 400x  B- Bcl-2 IHC stain 400x - showing Grade 3 positivity.

The highest number of cases collected were of moderately differentiated squamous cell carcinoma, amounting to 22 cases (55%), followed by well-differentiated squamous cell carcinoma, 15 cases(37.5%), and poorly differentiated squamous cell carcinoma, amounting to three cases(7.5%). In OSCC, the correlation between histopathological grading of OSCC and Bcl-2 expression showed higher Bcl-2 expression in poorly differentiated squamous cell carcinoma as compared to well-differentiated and moderately differentiated squamous cell carcinoma (Figures 1-3).

The difference between the Bcl-2 expressions of various grades of OSCC was statistically significant, with a p-value of 0.001 (Table 2).

**Table 2:** Correlation of Bcl-2 expression and histological grading of OSCC (n=40) WDOSCC- Well differentiated oral squamous cell carcinoma, MDOSCC- Moderately differentiated oral squamous cell carcinoma, PDOSCC- Poorly differentiated oral squamous cell carcinoma

Bcl2 expression	Histological grades							
	WDOSCC(n=15)		MDOSCC(n=22)		PDOSCC(n=03)		Chi-Square	p Value
	N	%	N	%	N	%		
Grade 0	10	66.70%	0	0.00%	0	0.00%	50.009	0.001
Grade 1	5	33.30%	14	63.60%	0	0.00%		
Grade 2	0	0.00%	8	36.40%	1	33.30%		
Grade 3	0	0.00%	0	0.00%	2	66.70%		
Total	15	100%	22	100%	3	100%		

**Figure 4:**
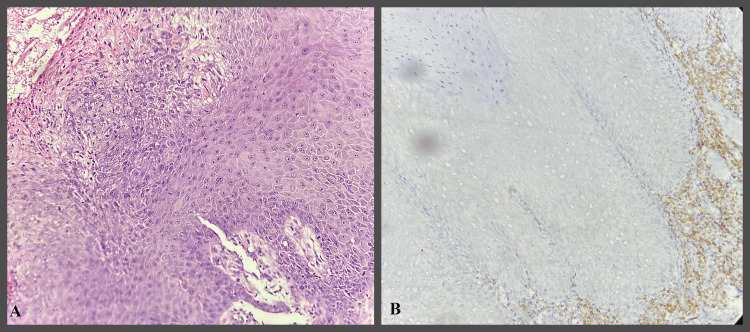
IHC study images showing mild epithelial dysplasia H&E- Hematoxylin and Eosin IHC- Immunohistochemical A- H&E stain 400x B- Bcl-2 IHC stain 400x - showing Bcl-2 expression in basal layer

**Figure 5:**
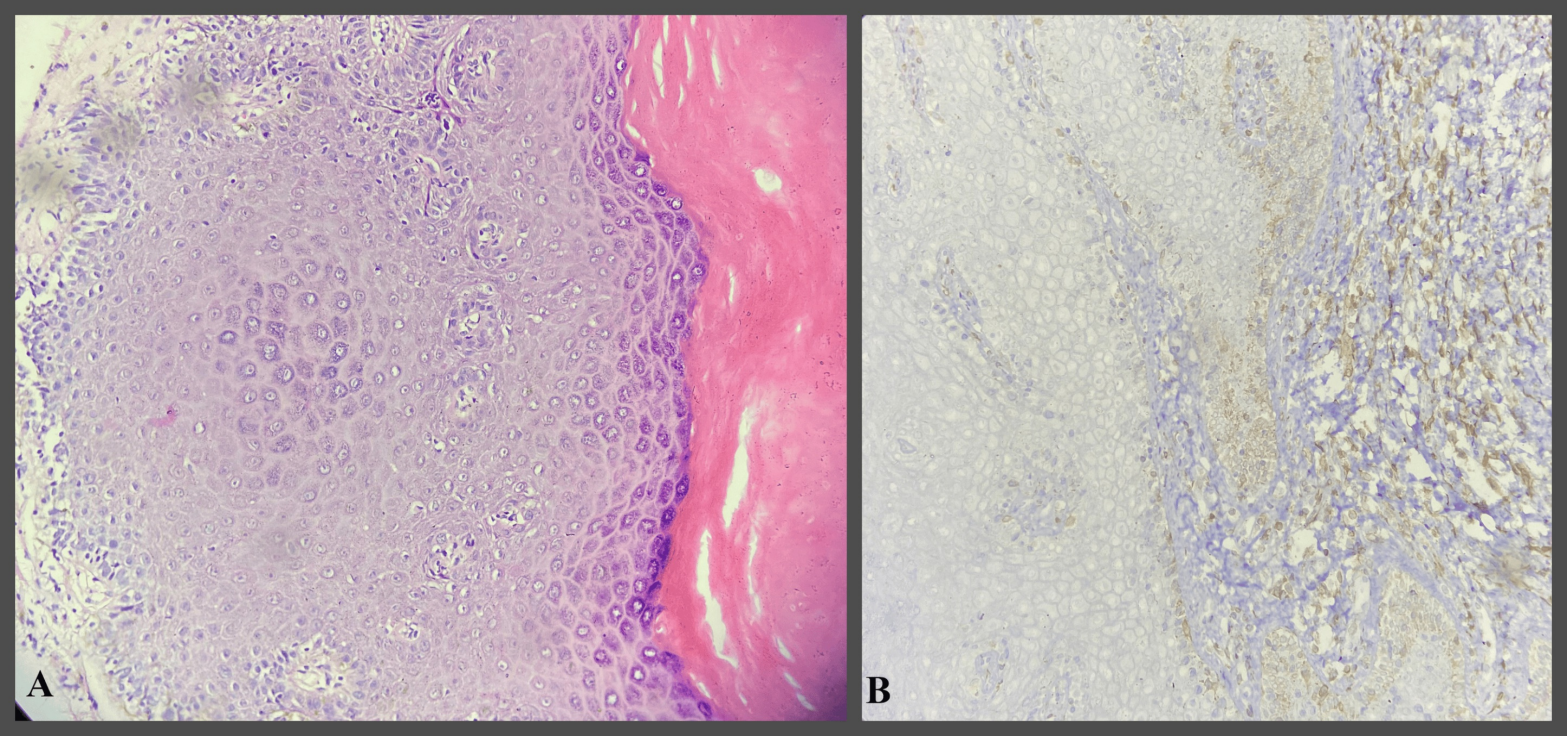
IHC study images showing moderate epithelial dysplasia H&E- Hematoxylin and Eosin IHC- Immunohistochemical A- H&E stain 400x B- Bcl-2 IHC stain 400x - showing Bcl-2 expression in basal and parabasal layers.

**Figure 6:**
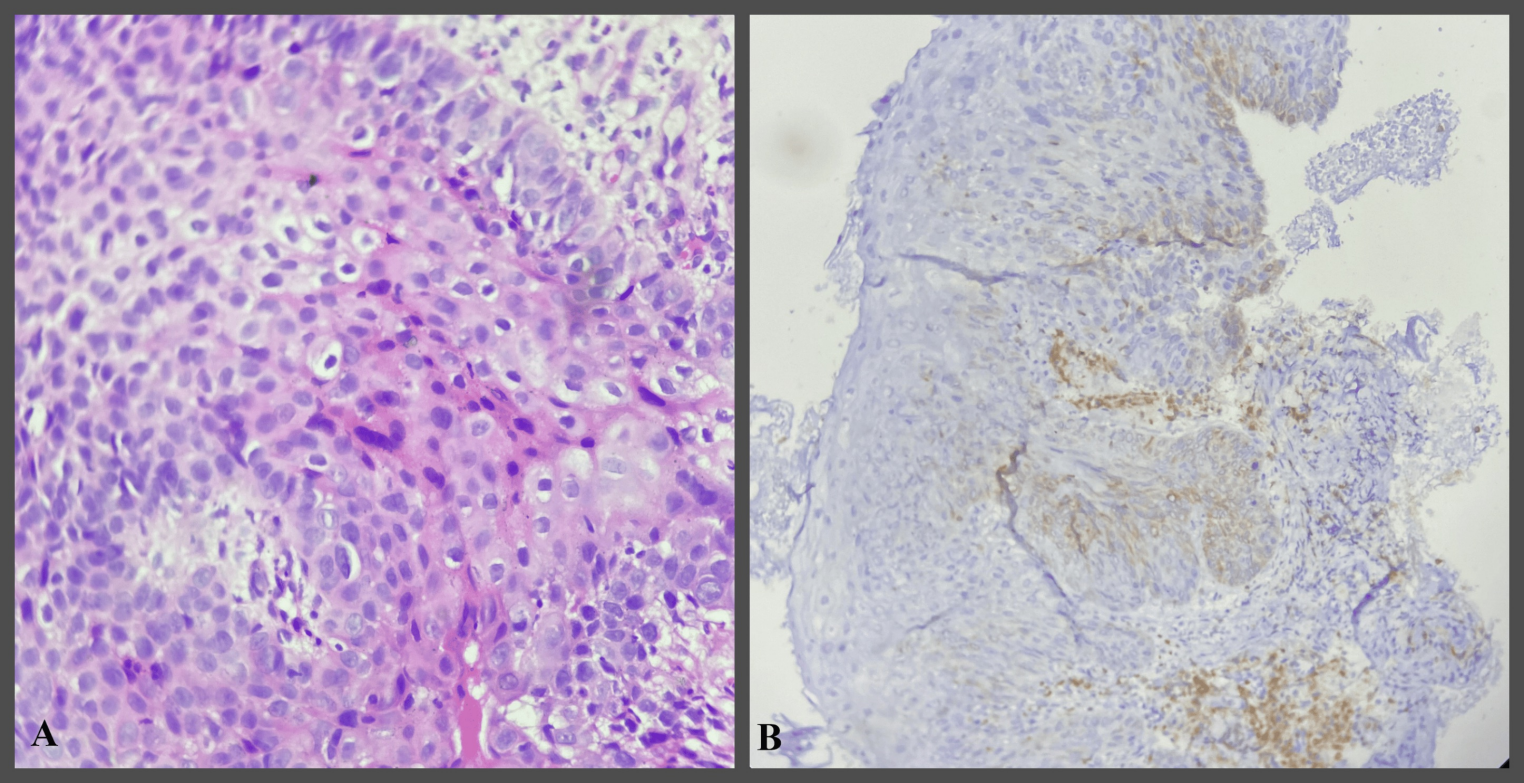
IHC study images showing severe epithelial dysplasia H&E- Hematoxylin and Eosin IHC- Immunohistochemical A- H&E stain 400x B- Bcl-2 IHC stain 400x- showing Bcl-2 expression in basal and suprabasal layers.

In OED, the highest number of cases observed were of mild dysplasia, amounting to 18 cases(45%), followed by moderate dysplasia, amounting to 11 cases (27.5%) and severe dysplasia, amounting to 11 cases(27.5%). The age group of 41-50 years showed the highest number of cases, amounting to 10 cases(25%), with a male preponderance of 8 (29.7%) (Table 3). Maximum number of cases of severe dysplasia were noted in the age group of 51-80 years. Severe dysplasia showed Bcl-2 expression in all three layers of lining epithelium. In moderate dysplasia, Bcl-2 expression was noted in the basal and parabasal layers, and mild dysplasia showed Bcl-2 expression only in the basal layer (Figures 4-6). Grade 2 expression was seen in eight cases (72.7%) cases of severe dysplasia and seven cases(63.6%) of moderate dysplasia. Of the 17 cases, none showed grade 2 expression in mid-dysplasia. Thus, the correlation between histopathological grading of dysplasia and Bcl-2 expression showed higher Bcl-2 expression in severe dysplasia compared to moderate and mild dysplasia. The difference in Bcl-2 expression in various grades of dysplasia was statistically significant, with a p-value of 0.001 (Table 4).

**Table 3:** Distribution of age and sex in OED (n=40) OED- Oral epithelial dysplasia

Age (in years)	Males		Females		Total	
	N	%	N	%	N	%
11-20	1	3.7%	0	00%	1	2.5%
21-30	5	18.5%	2	15.4%	7	17.5%
31-40	5	18.5%	0	00%	5	12.5%
41-50	8	29.7%	2	15.4%	10	25%
51-60	3	11.1%	4	30.8%	7	17.5%
61-70	4	14.8%	1	7.6%	5	12.5%
71-80	1	3.7%	4	30.8%	5	12.5%
Total	27	100%	13	100%	40	100%

**Table 4:** Correlation of Bcl-2 expression in OED with histological grading (n=40) OED- Oral epithelial dysplasia

Bcl-2 expression	Histological grades of epithelial dysplasia							
	Mild(n=18)		Moderate(n=11)		Severe(n=11)		Chi-Square	p Value
	N	%	N	%	N	%		
Grade 0	17	94.40%	0	0.00%	0	0.00%	36.813	0.001
Grade 1	1	5.60%	4	36.40%	3	27.30%		
Grade 2	0	0.00%	7	63.60%	8	72.70%		
Total	18	100%	11	100%	11	100%		

## Discussion

A detailed understanding of the molecular mechanisms causing OSCC is crucial for early detection and intervention, leading to improved patient survival rates [7,8]. In tumorigenesis, a critical step is the suppression of apoptosis, represented by increased Bcl-2 expression [9]. High expression of Bcl-2 has shown significant predictive value for chemotherapy response. Some studies indicated that lower Bcl-2 expression correlates with a better prognosis, while higher expression is linked to a poorer outcome. Reduced Bcl-2 levels are typically accompanied by increased Bax protein expression, which promotes apoptosis [10,11].

In a study by Bhutani et al., the average patient's age ranged between 34 and 85 years [12]. Across several studies, the age range between 41 and 70 was the most common for OSCC presentations [1,2,12-14]. The age range for OSCC in the present study correlates with the findings of other authors. In a study done by various authors, it was observed that most of the cases of OSCC were males, ranging from 60% to 83.3% [1,2,12,13,15,16]. Similar observations were noted in the present study, where 54 cases were males, amounting to 67.5%.

In the study done by Bhutani et al., the tongue was the most common site (61.3%), followed by the tonsil (24%) [12]. In the study done by Arya et al., the commonest site of malignancy in the oral cavity was buccal mucosa (58.6%), followed by the tongue (17.6%), alveolus (13%) and floor of mouth (10.8%) [2]. In a study by Jairajpuri et al., the most common OSCC subsites were the tongue and buccal mucosa, 38.7% and 28.9%, respectively [15]. Similar findings were observed in the current study, where the tongue was the commonest site, amounting to 21 (52.5%), followed by the buccal mucosa, amounting to 11 (27.5%), and gingivobuccal sulcus, amounting to two (5%). The percentage of the other affected sites, like the angle of the mandible, hard palate and angle of the mouth, were three (7.5%), two (5%) and one (2.5%), respectively.

In the study by Jones et al., when a correlation was done between age and Bcl-2 expression, high Bcl-2 expression was noted in the age group of more than 50 years [16]. Patients under 50 showed low Bcl-2 expression, and more than 50 showed high Bcl-2 expression, amounting to 54.4% [12]. They also conducted a correlation between Bcl-2 expression and gender using the Chi-square test, where it was observed that Bcl-2 expression was higher in males than females. However, the association between Bcl-2 expression and the patient's age was insignificant; however, the association between Bcl-2 expression and gender was statistically significant in OSCC. Similar findings were observed in our study, with slightly higher Bcl-2 expression in males 27 (67.5%) than in females 13 (32.5%). However, in the present study, the difference was statistically not significant.

In various studies, the expression of Bcl-2 was increased with increasing tumor grades [14,16]. In the present study, poorly differentiated oral squamous cell carcinoma showed higher (Grade 3) positivity 2 (66.70%) than moderately differentiated oral squamous cell carcinoma and well-differentiated oral squamous cell carcinoma. Most of the cases of well-differentiated and moderately differentiated oral squamous cell carcinoma showed Grade 1 positivity amounting to 5 (33.30%) and 14 (63.60%), respectively (Table 5). Similar findings were noted in studies done by Jones et al. [16] and Varshney et al. [14].

**Table 5:** Comparison of Bcl2 grading in OSCC with other studies WDOSCC- Well Differentiated Oral Squamous Cell Carcinoma MDOSCC- Moderately Differentiated Oral Squamous Cell Carcinoma PDOSCC- Poorly Differentiated Oral Squamous Cell Carcinoma.

Grades of OSCC	Jones et al.study[16]			Varshney et al. study [14]			Present study		
	Grade1	Grade2		Grade1	Grade2	Grade 3	Grade1	Grade2	Grade 3
WDOSCC	72.20%	14.20%	0.00%	12.50%	56.25%	0.00%	33.30%	0.00%	0.00%
MDOSCC	0.00%	71.40%	0.00%	22.22%	33.33%	27.77%	63.60%	36.40%	0.00%
PDOSCC	0.00%	14.20%	100%	0.00%	0.00%	100%	0.00%	33.30%	66.70%

Various studies suggest that the variations in patterns of Bcl-2 expression might be associated with alternative mRNA splicing of the Bcl-2 protein in many tumors. It is noted that Bcl-2 protein isoforms are present in cell lines and human tissues. Additionally, the differences in Bcl-2 expression patterns observed with Bcl-2 antibodies could be explained by selective caspase activation that leads to proteolytic degradation of Bcl-2 in tumor cells. Thus, it is worth considering that potential Bcl-2 isoforms could affect the sensitivity and specificity of the current Bcl-2 antibodies [17,18]. In a study by Arya et al., out of 16 oral epithelial dysplasia cases, 8% showed low Bcl-2 expression, 6% showed moderate expression, and none showed high expression. The remaining 2% of cases showed negative expression [2]. In a study by Nitya et al., Bcl-2 expression was noted in 24.73% of cases of various degrees of oral epithelial dysplasia [19]. Similar findings were noted in the present study. Of the 11 cases of severe dysplasia, 72.70% showed Grade 3 Bcl-2 expression, which is higher than mild and moderate epithelial dysplasia. The limitation of the present study was that the sample size of poorly differentiated oral squamous cell carcinoma is very small. Hence, a multi-centric study with more cases may help conclude the role of Bcl-2 expression as a prognostic marker.

## Conclusions

Increased Bcl-2 expression was found in all the layers in severe dysplasia. It may be due to the higher anti-apoptotic activity in severe dysplastic lesions, which demonstrates the aggressive nature of the lesion and high proliferative activity. Bcl-2 expression was also significantly higher in poorly differentiated oral squamous cell carcinoma than well and moderately differentiated oral squamous cell carcinoma, indicating that Bcl-2 expression increases with decreasing tissue differentiation, indicating the aggressive nature of the lesion and high proliferative activity.

Thus, the histological grading of oral squamous cell carcinoma and the degree of oral epithelial dysplasia can be predicted and prognostically assessed using Bcl-2. Furthermore, Bcl-2 has the potential to serve as a prognostic indicator for the identification of malignant transformation in epithelial dysplasia. Additionally, it can support early diagnosis of oral premalignant and malignant lesions, improving the patient's prognosis and allowing for early treatment. Since Bcl-2 is an anti-apoptotic marker, it can be helpful as a prognostic marker. It may further help evaluate the treatment of patients with oral epithelial dysplasia and oral squamous cell carcinoma.
